# Parents’ attitudes towards the No Jab No Play legislation in Western Australia: a mixed methods study

**DOI:** 10.1186/s12889-024-18995-9

**Published:** 2024-06-05

**Authors:** Sharyn Burns, Ranila Bhoyroo, Justine E. Leavy, Jonine Jancey, Hanna Saltis, Lynne Millar, Jacqueline Hendriks, Linda Portsmouth, Jenny Tohotoa, Danveer Seewoo, Christina Pollard

**Affiliations:** 1 Collaboration for Evidence, Research and Impact in Public Health, School of Population Health, Curtin University, Bentley, WA 6102 Australia; 2 Telethon Kids Institute, Perth, WA 6102 Australia

**Keywords:** Childhood vaccination, Childhood immunisation, No Jab No Play, No Jab No Play Policy, Vaccine mandates, Financial impact, Emotional impact, Educational impact, Parent attitude

## Abstract

**Background:**

Mandates provide a relatively cost-effective strategy to increase vaccinate rates. Since 2014, five Australian states have implemented No Jab No Play (NJPlay) policies that require children to be fully immunised to attend early childhood education and childcare services. In Western Australia, where this study was conducted, NJNPlay legislation was enacted in 2019. While most Australian families support vaccine mandates, there are a range of complexities and unintended consequences for some families. This research explores the impact on families of the NJNPlay legislation in Western Australia (WA).

**Methods:**

This mixed-methods study used an online parent/carer survey (*n* = 261) representing 427 children and in-depth interviews (*n* = 18) to investigate: (1) the influence of the NJNPlay legislation on decision to vaccinate; and (2) the financial and emotional impacts of NJNPlay legislation. Descriptive and bivariate tests were used to analyse the survey data and open-ended questions and interviews were analysed using reflexive thematic analysis to capture the experience and the reality of participants.

**Results:**

Approximately 60% of parents intended to vaccinate their child. Parents who had decided not to vaccinate their child/ren were significantly more likely to experience financial [*p* < 0.001] and emotional impacts [*p* < 0.001], compared to those who chose to vaccinate because of the mandate. Qualitative data were divided with around half of participants supporting childhood immunisation and NJNPlay with others discussing concerns. The themes (a) *belief in the importance of vaccination* and *ease of access*, (b) *individual and community protection*, and (c) *vaccine effectiveness, safety and alternatives* help understand how parents’ beliefs and access may influence vaccination uptake. Unintended impacts of NJNPlay included: (a) *lack of choice, pressure and coercion to vaccinate*; (b) *policy and community level stigma and discrimination*; (c) *financial and career impacts*; and (d) *loss of education opportunities.*

**Conclusions:**

Parents appreciation of funded immunisation programs and mandates which enhance individual and community protection was evident. However for others unintended consequences of the mandate resulted in significant social, emotional, financial and educational impacts. Long-term evidence highlights the positive impact of immunisation programs. Opinions of impacted families should be considered to alleviate mental health stressors.

## Background

The National Immunisation Program Schedule (NIPS) outlines a series of immunisations provided at no cost to people of all ages living in Australia enrolled in Medicare (Australia’s universal healthcare system). The childhood vaccination schedule applies to infants and children from birth to 5-years [1]. In March 2023, 94.2% of 5-year-old Australians were considered fully vaccinated (i.e. up-to-date with all scheduled vaccinations) [2], falling short of the 95% target recommended for herd immunity [3]. While overall coverage is relatively high, Australia reports some areas with pockets of lower coverage, with regional variations by Primary Health Network areas ranging from 90.02 to 96.38% [2]. This variation has been attributed to low socio-economic status, age, and culture [4] and to lifestyle choices of some higher socio-economic groups [5, 6]. Access to coordinated health care services has also been found to reduce inequalities in vaccines coverage. For example, a comparative study of measles vaccination coverage between Western Australia and New South Wales suggested the presence of public health units and Aboriginal medical centres in remote regions positively impacted coverage compared to outer regional areas where these services were limited [4].

In Australia, community support for childhood vaccination is generally high [7]. However, a study of Australian parents (*n* = 452) found despite 92% of parents reporting their child was up-to-date with their vaccinations, 52% held concerns [8]. Parental concerns have focused on safety and effectiveness of vaccines [5, 8, 9], and government childhood vaccination mandates [5, 7].

Mandates provide a relatively cost-effective strategy to increase immunisation rates [10], and the Australian government has implemented some type of childhood immunisation mandate since 1998 [11]. Currently, these mandates include the Federally funded No Jab No Pay (NJNPay) policy whereby parents must vaccinate their children to receive financial benefits unless the child has an approved medical exemption [12, 13]. Additionally, No Jab No Play (NJNPlay) policies are implemented at a State and Territory level and restrict access to early childhood education for children who are not immunised or who do not have a relevant exemption.

Since 2014, NJNPlay policies have been implemented across five Australian states with slight variations [14, 15] requiring children aged ≤ 5 years to be fully immunised according to the childhood NIPS before they can attend early childhood education services, including kindergarten and childcare [12]. In Western Australia (WA), where this study was undertaken, the NJNPlay legislation was enacted in 2019, with incorporated legislative changes to the Public Health Act (2016) and the School Education Act 1999 (WA)[16].

While the public health evidence for immunisation is unequivocal [13, 17], it is important to consider the impacts of mandates, including their unintended consequences. Although mandates are generally supported in Australia, debates around collective benefits versus individual choice highlight the need for ongoing evaluation [18–20]. A recent review of the literature found only two studies that specifically evaluated the NJNPlay legislation in isolation from NJNPay legislation [21]. This research aims to explore the impact of the NJNPlay legislation on families in WA, two years after its implementation.

## Methods

This mixed methods study was part of larger study conducted in WA. Data were collected via an online self-report survey and one-on-one phone interviews. The criteria for inclusion were parents or carers (hereafter parents) living in WA with at least one child aged ≤ 7 years.

### Recruitment and sample

Survey and interview participants were recruited via a convenience sample using a targeted social media strategy. The Australian Child Care Alliance and Playgroup WA supported recruitment and contacted relevant member organisations directly who disseminated recruitment materials (via social media posts, fliers, newsletters and email messages) to potential participants. Facebook posts were shared via relevant research project sites. A digital advertisement was placed on *Perth Now* (an interactive digital local news hub) for a period of 7.5 weeks.

Participants were provided an information sheet and consent was obtained from all participants before commencing the survey or interview. The surveys took approximately 15 minutes to complete and were open from 20 May to 5 September 2022. Survey participants were invited to enter a prize draw (3 x AUD$100 vouchers) and were also asked if they would like to participate in an interview at the conclusion of the survey. This information was removed from the data and stored securely as per the University HREC requirements. Parents who expressed interest in participating in an interview were contacted via email. Semi-structured, one-on-one interviews were conducted over the phone by one researcher, were audio-recorded, and lasted between 20 and 40 minutes. Participants received an AUD$30 gift card in appreciation of their time.

### Instrumentation

The larger study used a 29-item online survey. For the current analyses, a subset of data was derived from self-reported responses. Participants were asked questions regarding their sociodemographic status and the impact of the NJNPlay legislation on their family. Sociodemographic questions were based on standard questions used for the Australian Census [22]. Postcodes were used to determine participants’ region of residence. Closed and open-ended questions regarding the impact of NJNPlay were adapted from those used for an evaluation of NJNPlay in Victoria [23]. Surveys were tested for content validity with experts and face validity was established with a targeted sample (*n* = 5) of parents prior to administration.

The semi-structured one-on-one interview guide was informed by the literature [21] and preliminary analysis of the online survey data. Questions explored participants attitudes towards immunisation, the National Immunisation Program, the NJNPlay legislation and impacts of NJNPlay on their family. Open-ended questions in the survey built on closed responses to questions about attitude towards and impacts of NJNPlay and childhood immunisation.

### Data analysis

Descriptive statistics summarised the participant demographic characteristics (see Table 1). Associations were determined using Pearson chi-square with the binary variable parental intention to vaccinate their child/children and the following variables: influence of the NJNPlay legislation on decision to vaccinate; financial impact of NJNPlay legislation (impacted/not impacted and type of impact); and emotional impact of the NJNPlay legislation (impacted/not impacted and type of impact). Parents who indicated any financial and/or emotional impact of the legislation were asked if the impact was positive, mixed (i.e. some positive and some negative) or negative (see Table 2). Results with a p-value < 0.05 were considered statistically significant. Data were analysed in SPSS (version 27).

A reflexive thematic analysis approach was used to analyse qualitative data. A realist method was employed with the aim of reporting experiences and the reality of participants [24]. Braun and Clarke’s six phases were followed: data familiarisation; generating initial codes; generating initial themes; developing and reviewing themes; refining, defining and naming themes; and writing up [24].

The data analysis process, including transcription and theme formulation, was cross-referenced with the research team to maintain confirmability. Interviews were audio-recorded and transcribed verbatim. Line-by-line analysis was conducted to generate descriptive codes. Words and phrases were explored to determine shared meanings and perceptions. Codes were summarised into meaningful themes and original data reviewed against preliminary themes before final themes were defined and named. Transferability was ensured by providing detailed description of the research methods and results [25]. Transcriptions and coding were managed using the NVIVO V.12 software [26]. Data have been reported to discern between interview (PAR001) or survey participants (PS001).

## Results

### Survey results

The survey was completed by 261 parents, representing 427 children as some families had two or more children in the target age group. Demographics are described in Table 1. Sixty three percent of children were up-to-date with their immunisations based on parent/carer reporting. The definition of the NJNPlay legislation as relating to a child’s eligibility to enrol in childcare and/or kindergarten was correctly identified by 39.8% of participants. Over half of parents (56.3%) erroneously believed the legislation was related to financial incentives in addition to immunisation and enrolment eligibility.

**Table 1:** Demographic characteristics of survey participants

Characteristics	*n* (%)
**Residential location**	
Metropolitan	217 (83.1)
Regional (regional/rural/remote)	44 (16.9)
**Age**	
Under 24	3 (1.1)
25–29	19 (7.3)
30–34	75 (28.7)
35–39	98 (37.5)
40–44	47 (18.0)
45–49	16 (6.1)
Over 50	3 (1.1)
**Education**	
Did not complete high school	6 (2.3)
Completed high school	28 (10.7)
Completed TAFE/Trade	60 (23.0)
Completed a university degree	167 (64.0)
**Employment**	
Working full-time	56 (21.5)
Working part-time/casual	105 (40.2)
Home duties	89 (34.1)
Retired	1 (0.4)
Unable to work	2 (0.8)
Unemployed	2 (0.8)
Student	6 (2.3)
**Household income**	
Under $40,000	7 (2.7)
$40,001 - $100,000	75 (28.7)
$100,001 - $160,000	82 (31.4)
More than $160,000	67 (25.7)
Prefer not to answer	30 (11.5)
**Country born**	
Australia	190 (72.8)
Outside Australia	71 (27.2)
Card*	
A Health Care Card	31 (11.9)
Pension Concession Card	10 (3.8)
None of these	224 (85.8)
**Children care for aged 0–7 years**	
One [1]	125 (47.9)
Two [2]	108 (41.4)
Three [3]	26 (10.0)
Four [4]	2 (0.8)
**Children identified as Aboriginal or Torres Strait Islander**	
Yes	3 (1.1)
No	254 (97.3)
Prefer not to answer	4 (1.5)

* *Note* Participants could indicate one or more options

Almost 60% (*n* = 155) of parents indicated they intended to fully vaccinate their child. Of the 106 parents who were not intending/undecided to vaccinate 31 (29.3%) indicated the NJNPlay legislation influenced their decision to vaccinate in some way. Parents who had decided not to vaccinate their children were more likely to consider vaccination after the NJNPlay legislation was implemented compared with those who had already intended to vaccinate their children (*p* = 0.003). Participants were more likely to be impacted financially by the introduction of the legislation if they were not intending/undecided to vaccinate (*n* = 88; 82.2%) compared to those who were intending to vaccinate (*n* = 19; 17.8%). Of parents impacted financially (*n* = 107), 90.9% (*n* = 85) indicated the impact was negative. A total of 124 participants indicated they were emotionally impacted by the introduction of the legislation, with those not intending/undecided to vaccinate (*n* = 98; 79.0%) being more likely to be impacted compared to those who were intending to vaccinate (*n* = 26; 21.0%). For those impacted emotionally (*n* = 124), 68.5% (*n* = 85) indicated the impact to be negative. Parents who had decided not to vaccinate their child/ren were significantly more likely to experience financial [*p* < 0.001] and emotional impacts [*p* < 0.001], compared to those who chose to vaccinate (Table 2).

**Table 2:** Association between intention to vaccinate and perceived impact of NJNPlay legislation

	Were you intending on vaccinating your child/children aged 0–7 years?				
	Yes [n (%)]	No/Had not decided [n (%)]	Total (N)	Chi-Square	*p-value*
**Did the ‘No Jab No Play’ laws influence your decision to vaccinate your child/children aged 0–7 years?**				8.8	0.003
Yes/Partially	22 (41.5)	31 (58.5)	53		
No	133 (63.9)	75 (36.1)	208		
**The No Jab No Play legislation has affected my family financially**				130.3	< 0.001
Not impacted	136 (88.3)	18 (11.7)	154		
Impacted	19 (17.8)	88 (82.2)	107		
Type of financial impact* ^**†**^				6.4	0.011
Mixed (some positive and some negative)	7 (35.0)	13 (65.0)	20		
Negative	10 (11.8)	75 (88.2)	85		
**The No Jab No Play laws have affected my family emotionally**				144.6	< 0.001
Not impacted	129 (94.2)	8 (5.8)	137		
Impacted	26 (21.0)	98 (79.0)	124		
Type of emotional impact* ^**†**^				0.9	0.345
Mixed (some positive and some negative)	6 (25.0)	18 (75.0)	24		
Negative	16 (16.7)	80 (83.3)	85		

Note * = Positive responses less than 5 were excluded from the analyses.^†^ = Those who responded yes to being financially or emotionally impacted

### Qualitative results

Interview participants were almost all female (*n* = 17). Twelve were from the Perth metropolitan area and six from regional/rural WA areas. Of parents interviewed, half (*n* = 9; 50%) indicated their children were up-to-date with their vaccinations. Almost all (*n* = 259) parents provided responses to the open-ended questions in the survey.

Attitudes towards the NJNPlay legalisation and childhood immunisation were mixed with some participants being very supportive of the legislation and immunisation, while others expressed concerns and distrust.

Although parents were specifically asked about the impact of NJNPlay, they tended to discuss the interrelated issues of the NJNPlay and NJNPay with many confusing the legislations especially around payment of the Family Tax Benefit (an Australian government means tested assistance payment for families with children). A few participants were unclear about the scope of NJNPlay, with a small number of respondents assuming the legislation impacted all year levels of school enrolment. Findings are reported as contextual factors and unintended consequences of NJNPlay.

#### Contextual factors

The findings highlighted key **contextual factors** influencing participants beliefs and access to vaccination. Within contextual factors, the following themes were generated to help understand how beliefs and access may shape vaccination uptake; (a) *belief in the importance of vaccination* and *ease of access, (b) individual and community protection, and (c) vaccine effectiveness, safety and alternatives.*

##### Belief in the importance of vaccination and ease of access

Participants felt the immunisation schedule and immunisation services made it easy for parents to access vaccines and to know when their child was due to be immunised. Overall acceptance of the immunisation program was highlighted with some participants discussing the ease of access and others describing their amenability to vaccination in general, for example: “*happy to do it, always have been. I’ve never had any issues”* PAR008 and similarly *“my family was happy to vaccinate me and we didn’t have any adverse side effects that I’m aware of, so I think that probably helps, but it’s just part of our family values”* PAR005. Parents highlighted the importance of the schedule discussing access as well as protection from an individual and community perspective:

*“I am glad that it is mandated …on this schedule for two reasons. One, it makes it easier for parents like me to follow and essentially be told, you know, when vaccinations are due and what they are and two so that I know that a decent number of children out in society and you know, in education, are vaccinated and they’ll obviously be kids that my children are interacting with as well”* PAR012.

##### Individual and community protection

Positive responses to the NJNPlay legislation highlighted the benefits of protection and the achievements of immunisation. The following comment highlights this sentiment:

*“It is one of the most incredible developments in the 21st century that has saved countless lives and long-term medical conditions. People’s memories and understanding of life with polio, measles and other illnesses is far too short” (PS97).*

Some participants discussed protection from an individual perspective *“I think it’s great that we have these medicines that can stop our children getting preventable diseases”* PAR014, while others supported population level protection: *“I think it’s helpful to know that when you send your kids to school, that all the kids are going to be vaccinated as well because, you know herd immunity and those sorts of things is what protects the community”* PAR005. Parents discussed the benefits of the NJNPlay legislation in regard to protection for children and their families: *“I think it’s fair to require kids to be vaccinated before they go to educational facilities because it wouldn’t be fair for other kids and their parents to be put at risk: I support the no jab no play policy”* PAR012. Some participants also recognised the benefits of protecting those who were too young to be vaccinated and those older adults that interact with young children.

*We feel more secure with our kids playing and associating with others within the community as they are immunised against common serious virus/bacteria etcetera. “We live in an area with lower immunisation rates, so we feel a bit better when hearing there are vaccine preventable illness circulating within the community, that our kids and their friends are immunised. It is another layer of protection for our kids and by association grandparents. Our family is affected by being thankful for this legislation” (PS127).*

However, some participants did not agree that immunisation programs were beneficial at an individual or community level. Some felt concepts like herd immunity were misleading: *“I think herd immunity, vaccine-induced herd immunity is false; they haven’t actually done any studies, whether herd immunity exists because of vaccines or whether it exists through natural immunity”* PAR004. Some felt because of herd immunity, their children did not need to be vaccinated while others did not believe the legislation had any impact on immunisation rates.

*“If these vaccines are supposedly so effective in protecting my children from communicable diseases then why does it matter if a small portion of children aren’t vaccinated?” (PS213).*

*“I just don’t really understand what is the point in no jab no play to be honest, because I believe it’s to improve the rate of vaccination. But if you look then the rate has not increased. And I just think if it’s people like me, we’re just not gonna change our opinion over some legislation”* PAR007.

##### Vaccine effectiveness, safety and alternatives

Key to participants discussion was their perceived knowledge of childhood vaccinations and in particular the NIPS. Around half of participants were pro-vaccination and discussed the importance of immunisation and the values of the NIPS. A few participants felt the role of NJNPlay in promoting discussion about vaccination was important, for example: “*It’s [the NJNPlay legislation] started a healthy discussion around vaccines that may not occur otherwise”* (PS109).

Support was generally around the safety and effectiveness of vaccines, for example: “*I’m happy for the ones [vaccinations] I had as a kid, so my children, too, have [been vaccinated], because obviously there’s 40 years of documentation of side effects or issues”* (PAR011).

Participants were generally aware vaccination posed some risks however supportive parents considered the benefits of vaccination outweighed the risks associated with diseases. Parents recognised vaccines were beneficial in reducing the likelihood of contracting childhood diseases and in protecting against disease severity: “*Weighing up the benefits and risks, I suppose in my understanding is that it is better to vaccinate”* (PAR005).

Some participants, while supportive of having their child vaccinated portrayed some complacency. For example, when asked about what they thought of vaccination and the NIPS one participant stated: “*I wouldn’t actually have a clue, I just immunise them and get it over and done with”* (PAR008).

While around half of participants felt vaccines were safe and effective, the remaining half raised concerns around the efficacy and safety of vaccines. These concerns focused on the number of vaccines included on the schedule, negative side effects of vaccines and safety of vaccines. Some parents discussed specific examples of their child experiencing side effects, for example: “*She reacted so badly to a 12 month one. We ended up in the Perth Children Hospital emergency*” PAR018.

Others were sceptical around the safety and efficacy of vaccines, for example: *“I’m sceptical about having immunisations for everything and, yeah, I’m not sure about their safety or efficacy”* PAR007. Some participants were concerned vaccine testing was not rigorous and highlighted mistrust in pharmaceutical companies, for example: *“I’m not sure that it’s not just testing, trial testing, like I’m wanting to know where’s the proof?”* PAR011 and *If vaccinations are truly for the benefit of society and not for the profit of big pharmaceutical companies, the government and the elite, why would they not look at both the pros and cons in order to truly protect our most precious resources (i.e. children)? (PS241).*

Anxiety about the side effects of vaccines ranged from pyrexia and vomiting to serious anaphylaxis. Parents expressed their concerns about immature infant immune systems and the long-term effects of so many immunisations: *“Their immune systems and their bodies are just not developed to take on that many extra things, and particularly all the extra ingredients and stuff that are in the immunisations that can cause sometimes life threatening and lifelong things, going wrong.”* PAR004.The erroneous fear of autism was raised by several parents and influenced their decision not to immunise their children: “*In terms of autism, I do think that there is some sort of a link”* PAR002.

Some parents felt there were too many vaccines listed on the childhood immunisation schedule, for example: *“The amount of vaccines we are giving them is insane”* PAR002 and *“It’s quite a lot of immunisations that seems to happen quite frequently. It’s a lot more than what we had as kids”* PAR001 and *“I think it’s excessive given the improvement in medicine, technology, what we know about disease transmission”* PAR018.

A few parents questioned the reasons for vaccination of diseases that have been eradicated, for example: *“I just don’t know why we still use immunisation for ones that have been eradicated”* PAR009; and *“the diseases were pretty much non-existent and very minimal cases and no real deaths”* PAR017.

A few parents who did not vaccinate their children were enthusiastic about alternatives to protecting their children’s immune systems:” *I’ve got a lot of alternative therapies that if they do catch the childhood diseases, I would feel confident in being able to provide alternative therapies and treatments- I’ve found homeopathy and that gave me a little bit more confidence. So, I did homeopathic prophylaxis”* PAR003 and “*I immunise my children homoeopathically. I don’t use the vaccine, you know, the government vaccine schedule*” PAR010.

#### Unintended impacts of the NJNPlay legislation

While around half of the parents in this study were positive towards the NJNPlay legislation and appreciated the availability and protection vaccination provided, others discussed negative impacts. These interrelated subthemes impacted the social and emotional health of families, with some participants expressing concern for their mental health. Subthemes included: (a) *lack of choice; pressures and coercion to vaccinate*; (b) *policy and community level stigma and discrimination*; (c) f*inancial and career impacts*; and (d) *loss of educational opportunities*.

##### Lack of choice, pressure and coercion to vaccinate

Some parents discussed feeling they lacked control over their decision to vaccinate their child. While some participants had financial capacity to elect not to vaccinate their child, others felt they were coerced into vaccinating their child due to a lack of alternative options. Parents expressed concerns around the need to vaccinate so they could continue their career or to secure a place at an early childhood education centre. The following comments highlight lack of choice and perceived coercion:

*“I felt coerced into vaccinating my children to get them into kindy. My two older children both had bad reactions to the vaccines they received and still were not eligible for an exemption” (PS65).*

*“Coercion is not consent. Being forced to make a decision based on the fear you may not be able to afford food and rent is horrible and makes one feel powerless” (PS151)*.

*“Ethically, it’s completely wrong. It’s coercion. How is this legislation ethical? It’s created a lot of discussion in our family” (PS13)*.

For many feelings of coercion were directed at the government. Most who discussed government influences highlighted a lack of trust, for example: “*Major loss of trust in our government. Coercion is not consent”* (PS224) and expressed concerns around authoritarian approaches, for example:

*“It upsets me that we have a tyrannical government who uses financial coercion to inject children. It upsets me I can’t contribute to my profession and earn a significant living for our family. It feels not good to be unemployed or underemployed because of government discrimination relating to my child’s vax status” (PS227)*.

*“I have lost faith in all politicians and will never again vote for any party that supports this legislation. I think it is a breach of human rights to deny an early education to healthy children because of their vaccine status” (PS65).*

##### Policy and community level stigma and discrimination

Feelings of stigma and discrimination impacted some families, with some participants discussing mental health concerns. Some participants discussed the community-level stigma around not vaccinating children, while others suggested the policy directly impacted discrimination:

*“I think any policy that discriminates on people’s choice, that I think should be a free choice of what you do, whether you have vaccinations or not; to discriminate against someone for not, I find I’m offended by that”* PAR011.

*“It’s blackmail essentially”* PAR018.

Discrimination, marginalisation, social isolation and, for some, mental health concerns, were related to financial and educational impacts. Participants also expressed feelings of guilt associated with lost educational opportunities for their child.

*“The financial impact has affected us emotionally as well as the feeling of ostracism from society for not conforming to draconian laws” (PS20).*

*“I have felt extremely excluded. It has caused me and continues to cause me anxiety. I am hypersensitive in social situations when asked about my children attending kindergarten” (PS8).*

*“I feel guilty towards my child because she missed out on opportunities and early learning only because her parents made certain choices” (PS113).*

The complexity of mental health problems was evident. Parents discussed issues of social isolation, some of which were compounded by lack of career opportunities and financial pressures. Others discussed isolation in association with stigma and discrimination. The following highlights some of these complexities:

*“The emotional impact on me due to being forced out of my career and the social isolation created by 6 years of full-time household duties has been substantial, having a substantial, negative impact on my mental health. I am also acutely aware my career will not recover from such a lengthy absence. To attempt to compensate for the cessation of my income, my spouse has been forced to work an insane number of hours in overtime. I have spent much of the last 6 years parenting alone and isolated, with a spouse away at work for extensive periods. This has created further stress and isolation for me, been difficult for our young children to deal with, and left him constantly verging on burnout”* (PS182).

For a few participants, discrimination extended to their experiences with medical professionals. Parents discussed their disappointment at the judgement and lack of empathy for their decision not to immunise their children. *“I see the judgment [from my GP] - He just gave me a spiel about how, you know it’s safe and you know these things are rare and basically, he won’t see any child in his practice unvaxed”* PAR013. Similarly, another parent reflected: *“when I queried my daughter’s reaction, I was shut down. I was sort of told “Oh well, you know, she’ll be fine. It’s just a reaction”* (PAR018). The overwhelming feelings of stigma and personal discrimination are highlighted:

*“I feel so let down by our government for making what I think are the best choices for my children. I’m not an idiot, and it should never ever be a blanket rule for vaccinations. It makes me very sad these mandates are in place, it just makes me feel very strange, it’s a feeling I can’t describe and it’s not a nice one. I’ve gone to doctors to discuss [immunisation? ] and they are quite rude about it, and it just makes my stomach sink. Feeling anxious and quite down lately because of all this”* (PS14).

##### Financial and career impacts

The financial implications of NJNPlay were discussed in terms of lost income. Parents who were unable to access childcare, or were unable to afford private care, found work opportunities were impacted. This contributed to loss of income and impacted on long term career opportunities.

“*If you don’t get a subsidy, you can’t go [to daycare]. It’s like a no pay and a no play, financially it’s tough- if your kids can’t get into care, then your career is over”* PAR002.

*“Because of this law, I will not be able to return to work due to my son not being immunised as he won’t be able to attend childcare. I had to resign my long-term managing position of 15 years to look after my son at home. There should be options for individual child care centres to accept unvaccinated children. I’m sure many families would be able to go back to work to help our failing economy”* (PS246).

*“Reduced income as we couldn’t access childcare while the children were not fully vaccinated. Then financial burden of private nanny until we could no longer afford that either” (PS146).*

*“Have to pay full fee childcare ($150/day) as my daughter is not up-to-date with her vax schedule. She reacted so badly to previous vax (even when they were spaced out and not given all at one time) that we ended up in hospital emergency twice. How can I keep getting her jabbed when this is how she reacts? So, we have to pay full fee. It’s a complete rort”* (PS13).

The potential for inequity was also noted: *“Making it an all or nothing, one size fits all, everybody has to do this kind of thing and if not, you don’t get the same benefits, is very polarizing and it’s very difficult, and it’s just making that gap between, you know, the rich and poor even wider”* PAR004.

The legislation did provide some parents of non or partially vaccinated children the unexpected opportunity to spend more time with their children. However, participants who were able to stay at home acknowledged they were financially secure, for example:

*“I had been intending on having both children in full-time day-care. That would minimise interruption to my work. However due to the introduction of No Jab No Play, I ended up resigning from my position and looking after the children full-time. My husband earns quite a bit more than me so we decided that I would care for the children. This meant a huge drop in family income. Also, I am not getting any superannuation. And I am unsure about what kind of job I will be able to do when I re-enter the workforce because my skills will probably be out of date. But now I am looking after the children full-time. I actually have come to love it. My kids are really happy and doing really well, and I think it’s because I am there for them 100%”* (PS48).

##### Loss of education opportunities

Parents voiced their concern about their children not being able to attend an early childhood program. Discussion included concerns around lost educational and social opportunities with some parents worried about how their child would fare when they get to school, for example:

“*It doesn’t make sense to me to not have children mixing that are vaccinated or not vaccinated, so it affects us because my children by the time they get to pre-primary are disadvantaged because they haven’t been able to have that interaction with prekindy or kindy. And then they’re kind of thrown in the deep end*” (PAR003).

*“Due to medical reasons, my child is only partially vaccinated. Due to being only partially vaccinated, I work from home and my child has to be home with me. He has no friends and has next to no interaction with children his own age due to the no jab no play policy. My child is being penalised for something out of his control and its hard on him and hard on myself trying to meet his needs whilst juggling full time work”* (PS171).

*“I was really looking forward to taking my daughter to kindy and then for her to improve her social skills and her speech skills, which having cerebral palsy is very important, and her motor skills. So, they’re all the things preschool is all about preparing kids and yeah, so I was pretty sad”* PAR007.

Parents talked about their children being excluded based on their decision not to vaccinate: *“I think it’s a bit excessive and very exclusionary, if that’s even a word, excluding children from certain socialisation, learning experiences based on the decision of a parent”* PAR018.

While some parents struggled due to lack of access to early childhood education opportunities, some became creative providing their child with alternate ways to encourage socialisation. Although participants did recognise their social privilege enabled these opportunities: *“It’s more of the social aspect of things, you know, sharing toys and playing with other kids. So, we’re trying to take her to jungle gyms and things like that just so she can interact with the other kids”* PAR017.

*“We’ve been able to employ a nanny who is early childhood trained and we’re fortunate that our jobs are pretty well paying so we can afford that and we’ve been fortunate to find some, small home schooling programs”* PAR004.

## Discussion

This study adds to the small body of evidence that explores the impact of the NJNPlay legislation in WA. Findings suggest participants in this study were divided in their attitudes towards the NJNPlay legislation. Parents who were supportive discussed their belief in the importance of vaccination at an individual and population-based level. These participants were thankful the legislation was protecting their children and others. Similarly, other Australian studies have found parents do generally support childhood vaccines and the NJNPlay legislation [7] and parents generally trust and appreciate government funded vaccination programs [27].

However, similar to other Australian studies, some parents in this study expressed known concerns around immunisation, such as the number, spacing, safety of, and necessity for vaccines [5, 8, 9], especially in societies with few outbreaks of traditional childhood diseases [28]. While some participants supported population-based programs and understood the implications of herd immunity, others were sceptical. Despite long-term interventions to enhance community education [11], misinformation was provided by some participants in this study, specifically around the benefits of alternative therapies, hygiene and healthy living replacing the need to vaccinate and association with autism. These findings reinforce the need to dispel myths around vaccines, such as the disproven link to autism [29] while other conversations indicated a need for clarification around the safety, effectiveness, number and spacing of vaccines. Vaccines listed on the NIPS have undergone independent review by the Therapeutic Goods Administration and have met high efficacy, safety and quality standards [30]. Reliable data collected via the Australian Childhood Immunisation Register informs public policy, especially around vaccine safety and effectiveness [31].

However, mandated vaccination remains a much-debated ethical dilemma. A recent review highlights the importance of considering consequences for individuals compared to the collective, recognising that sometimes parental decisions not to vaccinate derive from concerns around vaccine safety and efficacy and potentially a personal or known negative experience. Consequences should be balanced against concerns around autonomy, consent and liberty, with refusal aligned with parental freedom of choice [32]. These findings are ratified by a study of four Australian community juries who supported mandates for childhood vaccination from the perspective of the collective, but recommended mandates be part of a co-ordinated intervention including a range of strategies and incentives. While both NJNPay and NJNPlay were supported, restricting access to financial incentives for parents were preferred to restricting access to a child’s education [18].

The sentiment that the NJNPlay legislation forced parents to immunise their child/ren was expressed by some participants. Feelings of coercion were associated with powerlessness and distrust for the government and pharmaceutical companies. In their study of non-vaccinating parents in Western Australia and South Australia, Attwell and colleagues (2017) found distrust to stem from the perception of the influence of pharmaceutical companies over research, health professionals and the government [33]. A perceived lack or choice and powerlessness manifested in mental health concerns for some parents, which was supported by the present survey data.

Social and emotional health issues were linked to social isolation and being ostracised. Community and medical stigma were identified in addition to concerns about being marginalised and discriminated against by friends, school families, the community, and by medical professionals, for choosing not to immunise their child. Another Australian study with non-vaccinating parents also found parents to experience ‘social othering’ and status loss and discrimination. Parents in this study expressed status loss in relation to their perceived mental and parental competence and provided examples at an interpersonal and systemic level [19]. The discrimination noted in this and other Australian studies [5, 23] may have been heightened by the media dialogue in Australia, which has vilified non-vaccinating parents and was instrumental in advocating for the NJNPlay legislation [14].

Similar to the Victorian evaluation [23] survey, our interview data highlighted the financial impacts of the legislation for some parents. Ineligibility to enrol in childcare resulted in lost parental career opportunities (usually affecting women), increased financial burden associated with loss of income, along with the need to secure alternative, and at times expensive childcare. Participants talked about the pressure to immunise their children, especially if it restricted their ability to work. These findings support those found in other qualitative studies with partially or non-vaccinating parents in Australia [5, 34].

Loss of educational opportunities for their child/ren resulted in guilt and remorse among parents. Participants were concerned their child/ren were being denied valuable socialisation and education opportunities and were potentially being discriminated against. It has been suggested that exclusion from child care or school is likely to have a class-based impact, distinguishing between those parents who can and cannot teach and care for their children at home [35]. Consistent with our study findings, these educational restrictions also highlighted the socio-economic inequities among parents, with some parents being able to afford alternate care such as a nanny and in-home educational opportunities while others could not. This suggests the burden may likely to be borne disproportionately by the economically and socially disadvantaged groups in our communities [35].

It is a global concern that results achieved by childhood immunisation are endangered by the growing phenomena of vaccine hesitancy and refusal [36, 37]. Western Australian studies in high-middle income areas found parents believe their place of residence resulted in reduced risk of contracting vaccine preventable diseases and positive health outcomes [9], with non-vaccinating and/or ‘vaccine hesitant’ parents being willing to accept the risks and the subsequent responsibility when choosing to abstain, or partially abstain, from vaccinating their children [34]. Like the current study, parents in these studies practised prevention strategies such as healthy diet and exercise, and homeopathy aimed at protecting and maintaining child health and reducing risk of infection and illness [9, 34]. However, there is no evidence in the academic literature of the effectiveness of these homeopathic treatments [38]. Australian parents have also been found to be concerned about being labelled as ‘anti-vaxers’ and were keen to discern themselves from parents who advocated for ‘alternative lifestyle choices’ suggesting their reasons were around being able to make a choice [19].

The growing infodemic, which has been exacerbated during COVID-19, may further contribute to disinformation and misinformation around vaccination [39, 40]. There is a critical need for a multi-strategy approach that includes clear targeted health communication messages using both traditional and digital media, increased education, skills and tools to discern, verify and disseminate sources of information regarding vaccination [41]. A recent review of childhood vaccination acceptance found some vaccine hesitant parents had a very sophisticated understanding of vaccines while some parents with limited knowledge were happy to vaccinate their children [42]. While population-based data indicates most parents do vaccinate their children, and as such comply with vaccine protocols, it is important to consider the perspectives of vaccine hesitant and refusing parents [33]. Vaccine hesitancy and refusal is complex and may be influenced by interrelated personal, social, political and philosophical reasons [37]. The World Health Organization has identified vaccine hesitancy as a major threat to global public health [43] and vaccine misinformation on social media is considered a significant contributor to this issue [44]. As public health professionals we need to recognise the need for open dialogue without discrimination.

Limitations to this study should be considered. Noteworthy, the NJNPlay legislation only relates to a child’s eligibility to enrol in childcare and pre-compulsory schooling while NJNPay relates to eligibility for family assistance payments. However, 56.3% of parents surveyed (n = 147) incorrectly thought NJNPlay related to both family assistance payments and enrolment eligibility. As a result, many parents discussed the effects of both legislations interchangeably. The use of a convenience sample resulted in the survey and the interviews attracting a higher proportion of partial or non-vaccinating parents and those with concerns about childhood vaccination when compared to the general population. Other studies have reported similar bias, for example, an Australian online survey found approximately 23% of participating parents indicated their child/ren under five years were not up-to-date with their vaccinations [7]. Over half (52.5%) of parent respondents to the Victorian NJNPlay evaluation reported their child/ren were unvaccinated and 16.8% reported partially vaccination 23. Participants were also more likely to be middle-high income and 64% had completed a university degree. Aboriginal and/or Torres Strait Islander people represent 3.3% of the Western Australian population however only 1.1% of participants in this study identified as Aboriginal and/or Torres Strait Islander [45]. Finally, this study was conducted during the COVID-19 pandemic which likely impacted on recruitment. The concurrent media attention to the COVID-19 vaccination program and the ‘infodemic’ of vaccine misinformation and disinformation [46] would likely have impacted findings.

Despite this, the sample provided useful insights into the unintended consequences of the NJNPlay legislation and enabled consideration of the viewpoints of parents who do not vaccinate their children. Standardised items used in other Australian evaluation research were used allowing State comparisons. The mixed methods approach allowed triangulation of data and to provide a richer analysis of impacts.

## Conclusions

Childhood immunisation coverage in Australia is high however a small percentage of parents elect not to vaccinate their children. This study highlights parents’ appreciation of funded immunisation programs and mandates such as NJNPlay which enhance individual and community protection. The findings do however highlight unintended consequences of the NJNPlay mandates for some families and the need to consider their opinions. While vaccine refusing families are unlikely to change their attitudes, findings from this study highlight the need for evidence-based education around the safety and efficacy of childhood vaccines, along with increased education around individual and community protection which may influence some vaccine hesitant parents. Public health interventions should consider issues of autonomy, consent and liberty and to further engage non-vaccinating parents in planning.

## Data Availability

The datasets used and/or analysed during the current study are available from the first author on reasonable request.

## Abbreviations

NIPS: National Immunisation Program Schedule (Australia)
NJNPay: No Jab No Pay legislation (Australia)
NJNPlay: No Jab No Play legislation (Australia)

## References

[1] Department of Health and Aged Care. National Immunisation Program Schedule. Australian Government. 2023. Updated 20 December 2022. Accessed 26/08/2023, 2023. https://www.health.gov.au/topics/immunisation/when-to-get-vaccinated/national-immunisation-program-schedule.

[2] Department of Health and Aged Care. Immunisation coverage rates for all children. Department of Health and Aged Care. 2023. Accessed 18 July 2023, https://www.health.gov.au/topics/immunisation/immunisation-data/childhood-immunisation-coverage/immunisation-coverage-rates-for-all-children#:~:text=At%20March%202023%2C%20the%20national,for%20all%20five%20year%20olds.

[3] National Centre for Immunisation Research and Surveillance. No jab no play, no jab no pay. 2020. Updated Updated May 2020.

[4] Arat A, Moore HC, Goldfield S, Ostberg V, Sheppeard V, Gidding HF. Childhood vaccination coverage in Australia: an equity perspective. BMC Public Health. 2021;21(1337). 10.1186/s12889-021-11345-z. 10.1186/s12889-021-11345-z PMC8261950 34229652

[5] Helps C, Leask J, Barclay L (2018). It just forces hardship: impacts of government financial penalties on non-vaccinating parents. J Public Health Policy.

[6] Kirby T (2017). No jab, no play: Australia and compulsory vaccination. Lancet Infect Dis.

[7] Trent MJ, Zhang EJ, Abrar Ahmad C, MacIntyre CR (2019). Parental opinions towards the No Jab, no pay policy in Australia. Vaccine.

[8] Chow MYK, Danchin M, Willaby HW, Pemeberton S, Leask J (2017). Parental attitudes, beliefs, behaviours and concerns towards childhood vaccinations in Australia: a national online survey. Australian J Gen Practitioners.

[9] Swaney SE, Burns S (2019). Exploring reasons for vaccine-hesitancy among higher‐SES parents in Perth, Western Australia. Health Promotion J Australia.

[10] Attwell K, Seth R, Beard F, Hendry A, Lawrence D. Financial interventions to increase Vaccine Coverage. Pediatrics. 2020;146(6). 10.1542/peds.2020-0724. 10.1542/peds.2020-0724 33199467

[11] NCIRS. Significant events in immunisation policy and practice. National Centre for Immunisation Research and Surveillance; 2018.

[12] Attwell K, Drislane S (2022). Australia’s ‘No jab no play’ policies: history, design and rationales. Aust N Z J Public Health.

[13] Beard FH, Leask J, McIntyre PB (2017). No jab, no pay and vaccine refusal in Australia: the jury is out. Med J Aust.

[14] Attwell K, Hannah A. Convergence on Coercion: functional and political pressures as drivers of Global Childhood Vaccine mandates. Int J Health Policy Manag. 2022;1–12. 10.34172/ijhpm.2022.6518. 10.34172/ijhpm.2022.6518 PMC9818102 35397484

[15] Attwell K, Navin MC, Lopalco PL, Jestin C, Reiter S, Omer SB (2018). Recent vaccine mandates in the United States, Europe and Australia: a comparative study. Vaccine.

[16] Jab N. No play to improve vaccination rates in WA Children. State Government of Western Australia; 2019.

[17] Baum F. The new public health. 4th ed. Oxford; 2016.

[18] Degeling C, Attwell K, Wood N (2023). Public values to guide childhood vaccination mandates: a report on four Australian community juries. Health Expect.

[19] Wiley K, Leask J, Attwell K (2021). Stigmatized for standing up for my child: a qualitative study of non-vaccinating parents in Australia. SSM - Popul Health.

[20] Wiley K, Robinson P, Degeling C, Ward P, Leask J, Carter S (2022). Get your own house in order’: qualitative dialogue groups with nonvaccinating parents on how measles outbreaks in their community should be managed. Health Expect.

[21] Burns S, Bhoyroo R, Leavy JE (2023). The impact of the No Jab No Play and no jab no pay legislation in Australia: a scoping review. Int J Environ Res Public Health.

[22] Australian Bureau of Statistics. Australian Bureau of Statistics 2019 4363.0 - National Health Survey: Users’ Guide, 2017-18 Australian Government. 2019. Accessed 8 August 2022, 2023. abs.gov.au/ausstats/abs@.nsf/Lookup/by%20Subject/4363.0 ~ 2017-18 ~ Main%20Features ~ Demographics%20characteristics ~ 52.

[23] Centre for Evaluation and Research Evidence. No jab no play 2020 review. Department of Health and Human Services; 2020.

[24] Braun V, Clarke V. Thematic analysis: a practical guide. SAGE; 2022.

[25] Liamputtong P. Qualitative research methods. 4th ed. Oxford University Press Australia; 2013.

[26] NVIVO, Version. 20. 2022. www.qsrinternational.com/nvivo/nvivo-products/nvivo-12-windows.aspx.

[27] Burns S, Roux F, Selvey L. Influences to HPV completion via a school-based immunisation program. Sex Educ. 2020;1–16. 10.1080/14681811.2020.1788527.

[28] Diekema DS (2012). Improving childhood vaccination rates. N Engl J Med.

[29] DeStefano F, Price CS, Weintraub ES (2013). Increasing exposure to antibody-stimulating proteins and polysaccharides in vaccines is not associated with risk of autism. J Pediatr.

[30] Lewis G, McGuire R. Devlopment and regulation of vaccines. Melbourne Vaccine Educaiton Centre; 2023. https://mvec.mcri.edu.au/references/development-of-vaccines/.

[31] Kpozehouen EB, Heywood AE, Menzies R, Seale H, Brotherton J, Macintyre CR (2023). Informing the design of a whole of life immunisation register for Australia. Vaccine.

[32] Wiley K, Christou-Ergos M, Degeling C, et al. Childhood vaccine refusal and what to do about it: a systematic review of the ethical literature. BMC Med Ethics. 2023;24(96). 10.1186/s12910-023-00978-x. 10.1186/s12910-023-00978-x PMC10633934 37940949

[33] Attwell K, Leask J, Meyer SB, Rokkas P, Ward P (2017). Vaccine rejecting parents’ Engagement with Expert systems that inform Vaccination Programs. Bioethical Inq.

[34] Ward PR, Attwell K, Meyer SB, Rokkas P, Leaske J (2018). Risk, responsibility and negative responses: a qualitative study of parental trust in childhood vaccinations. J Risk Res.

[35] Attwell K, Navin MC (2019). Childhood vaccination mandates: scope, sanctions, Severity, selectivity, and salience. Milbank Q.

[36] McIntosh E, Janda DG, Ehrich J, Jochen HH, Pettoello-Mantovani M, Somekh E (2016). Vaccine hesitancy and refusal. J Pediatr.

[37] Cooper S, Wiysonge CS (2023). Towards a more critical Public Health understanding of Vaccine Hesitancy: key insights from a Decade of Research. Vaccines.

[38] National Health and Medical Research Council. NHMRC Information Paper: evidence on the effectiveness of homeopathy for treating health conditions. National Health and Medical Research Council; 2015.

[39] Islam MSKA-H, Kabir A, Southern DL, Khan SH, Hasan SMM. al. e. COVID-19 vaccine rumors and conspiracy theories: the need for cognitive inoculation against misinformation to improve vaccine adherence. *PLoS One*. 2021;16(5):e0251605. 10.1371/journal.pone.0251605 PMC8115834 33979412

[40] White B, Burns SK, Carson J, Scott JA. Mapping breastfeeding and COVID-19 related content and engagement on Facebook: results from an online social listening study. Health Promotion J Australia. 2023:1–9. 10.1002/hpja.729. 10.1002/hpja.729 37076784

[41] Domgaard S, Park M (2021). Combating misinformation: the effects of infographics in verifying false vaccine news. Health Educ J.

[42] Cooper SS, Sambala B-M, Swartz EZ, Colvin A, Leon CJ, Wiysonge N. C.S,. Factors that influence parents’ and informal caregivers’ views and practices regarding routine childhood vaccination: a qualitative evidence synthesis. Cochrane Database Syst Rev 2021;10, CD013265. 10.1002/14651858.CD013265.pub2 PMC8550333 34706066

[43] World Health Organization (WHO). Ten health issues WHO will tackle this year. World Health Organization. 2019. https://www.who.int/news-room/spotlight/ten-threats-to-global-health-in-2019. Accessed 14 September, 2023.

[44] Borges do Nascimento IJ, Pizarro AB, Almeida JM (2022). Infodemics and health misinformation: a systematic review of reviews. Bull World Health Organ.

[45] Australian Bureau of Statistics. Where Aboriginal and Torres Strait Islander people live. 2022. Accessed 14/03/2024. 2024. Retrieved from: https://www.abs.gov.au/articles/western-australia-aboriginal-and-torres-strait-islander-population-summary#where-aboriginal-and-torres-strait-islander-people-live.

[46] Li YJ, Marga JJ, Cheung CMK, Shen XL, Lee MKO (2022). Health misinformation on social media: a systematic literature review and future research directions. AIS Trans Hum Comput Interact.

